# Occupational therapists' perceptions of artificial intelligence: Potential, preparedness, and training needs

**DOI:** 10.1111/1440-1630.70090

**Published:** 2026-05-05

**Authors:** Claire del Rio, Helen Nelson, Danielle Hitch

**Affiliations:** ^1^ Western Health Footscray Victoria Australia; ^2^ Western Health Sunshine Victoria Australia; ^3^ Deakin University Geelong Victoria Australia

**Keywords:** AI literacy, artificial intelligence, digital health, ethical use of technology, occupational therapy, workforce preparedness

## Abstract

**Introduction:**

Artificial intelligence (AI) is transforming health care, yet its impact on occupational therapy remains underexplored. This study investigated occupational therapists' perceptions of AI's potential, preparedness, and training needs and differences across therapist groups in a tertiary health service.

**Methods:**

A cross‐sectional online survey was conducted with 53 occupational therapists, including the Shinners Artificial Intelligence Perception tool and the AI Literacy Scale. Quantitative data were analysed descriptively and with exploratory inferential statistics, with content analysis applied to qualitative responses.

**Consumer and Community Involvement:**

There was no consumer and community involvement in this study.

**Findings:**

Participants reported low AI knowledge (M = 1.32), skills (M = 1.21), and confidence (M = 1.30). They acknowledged the potential for AI to enhance care but perceived limited preparedness for its implementation (M = 2.45), particularly in relation to training gaps and ethical concerns. Knowledge, skills, and confidence were strongly correlated, but service area or years of experience did not exert any significant influence. Participants wanted training about occupational therapy applications and how to use AI ethically and efficiently.

**Conclusion:**

Occupational therapists are optimistic about AI but need targeted education to enhance their literacy and practice, integrated within overall workforce development strategies.

## PLAIN LANGUAGE SUMMARY

This study asked occupational therapists at a big Australian hospital what they think about using artificial intelligence (AI) in their job. We asked 53 therapists questions about their AI knowledge and what training they want. Most therapists have almost no experience with AI. They say their knowledge, skills, and confidence are low, but they are excited about AI. Therapists who knew more about AI also felt more confident using it, but where they worked or their years of experience did not make much difference. They think AI could help patients by making assessments easier and reducing paperwork. They are worried about ethical problems and feel unprepared because they have had little training. Therapists want clear, practical training and simple workplace rules. This will help them use AI safely and well, so they can keep helping people live better, more meaningful lives.

**Key Points for Occupational Therapy**

- Occupational therapists want to use artificial intelligence in practice but lack the knowledge and confidence to do so.
- Targeted AI training and clear workplace policies will support ethical and effective AI use.
- Workforce development should prioritise building AI literacy within clinical settings.

## 1 INTRODUCTION

Artificial intelligence (AI) is increasingly shaping many aspects of modern life, including health care, where it has been promoted as a potential solution to workforce shortages, rising costs, and demand for more personalised care (Khanam et al., 2024; Meskó et al., 2018). AI performs tasks traditionally requiring human intelligence, such as learning, reasoning, and problem‐solving (Hitch, 2023). Subfields such as machine learning, deep learning, and reinforcement learning enable systems to identify patterns in complex data (Bajwa et al., 2021). In health care, these capabilities are already being applied to diagnostic imaging and predictive analytics and increasingly to data from wearable devices, with growing but variable evidence of efficiency and accuracy (Ghassemi et al., 2019).

Evidence about AI in occupational therapy is emerging but remains limited. Emerging studies have explored the potential of AI in occupational therapy‐relevant domains, including motor recovery, developmental milestones, and occupational engagement (Fair‐Field & Modayur, 2025; Kim et al., 2020; Kokkotis et al., 2025; Zhao & Zariffa, 2024). However, much of this work remains developmental or experimental, with limited translation into routine clinical practice. Currently, machine learning, robotics, and AI‐enabled decision‐support systems are the most common AI technologies used for occupational therapy interventions (Kansizoglou et al., 2025). Several descriptive and pilot studies suggest potential benefits for tailoring occupational therapy interventions, although this evidence also remains preliminary and contextually bound (Değerli & Özata Değerli, 2025; Jain et al., 2020; Lauer‐Schmaltz et al., 2024). AI technologies for reducing administrative burden, supporting documentation, and delivering personalised feedback are also being explored as a means of enhancing therapeutic interventions (Stover & Jacobs, 2025) and releasing time back to patient care.

Although it is in an early stage of adoption in occupational therapy, there is growing recognition that AI will likely have a profound effect on professional practice. However, the integration of AI into practice raises important ethical questions about client‐centred care, occupational justice, and professional values, necessitating careful reflection on how these tools can support rather than undermine the unique contribution of occupational therapy to human health (Kaelin et al., 2024). Realising AI's transformative potential in occupational therapy hinges on equipping occupational therapists with the skills and knowledge to integrate these technologies into practice. Early indications suggest that occupational therapy students and early‐career professionals will play a key role in AI implementation, as they are anticipated to be the first generation to develop their practice within the context of these rapidly developing technologies (Stover & Jacobs, 2025).

Research indicates that health‐care professionals generally report limited knowledge and skills for AI but feel both optimism and concern about its ethical use and impact on professional roles (Akhtar et al., 2025; Hoffman et al., 2024; Shinners et al., 2023; Vanamali et al., 2025). A recent Canadian study of occupational therapists identified variable understanding, but participants were not overly concerned about job displacement (Matharu et al., 2025). However, little is known about occupational therapy workforce preparedness in other contexts. Understanding occupational therapists' perceptions of AI is essential for workforce development, as these perceptions influence uptake, ethical engagement, and integration into daily practice. Further, a greater understanding of the relationships between AI literacy components may help identify leverage points for targeted workforce development.

The aim of this study was to explore the perceptions of occupational therapists of AI, including their AI literacy, preparedness for practice, perceived professional impact, and training needs within an Australian metropolitan health service. The research questions were as follows:

1. What are occupational therapists' levels of AI knowledge, literacy, confidence, and preparedness?
2. How do occupational therapists perceive the professional impact of AI?
3. Do perceptions differ by service setting, years of experience, or prior AI exposure?
4. What is the relationship between AI knowledge, skills, confidence, and comfort?

## 2 METHODS

A cross‐sectional, descriptive mixed‐methods design was used to investigate occupational therapists' knowledge, perceptions, and training needs regarding AI in health care.

### 2.1 Positionality statement

The research team comprised three clinician researchers with expertise in acute hospital service settings and workforce development. The first two authors (C. d. R. and H. N.) had novice knowledge and experience of AI, whereas the third author (D. H.) had undertaken higher education micro‐credentials in the field. All were committed to advancing understanding of AI in occupational therapy but acknowledged their personal interest in digital innovation. Reflexivity was maintained through iterative peer review and pilot testing of the survey instrument and regular data analysis discussions to minimise interpretive bias. D. H. led the data analysis, with C. d. R. and H. N. independently reviewing coding decisions and contributing to reflexive discussions throughout the analysis.

### 2.2 Participants and recruitment

Participants were Australian Health Practitioner Regulation Agency (AHPRA)‐registered occupational therapists employed at a large metropolitan health service in Melbourne, Australia. All staff were invited regardless of experience or clinical area. Recruitment occurred via staff emails, newsletters, and professional networks. The survey remained open for 6 weeks, with reminders every 2 weeks. At the time of the study, approximately 120 eligible occupational therapists were employed at the study site.

### 2.3 Survey instrument

A bespoke brief (5–7 minute) survey was developed using established frameworks and measures. It began with demographic questions about gender, age, service area, years of practice, and years of AI experience. Self‐rated competencies in knowledge, skills, confidence, and comfort with AI were assessed using single items on 5‐point Likert scales, ranging from *poor* to *excellent*. This scale was selected to provide a simple, intuitive self‐assessment of perceived competence, consistent with exploratory workforce surveys. These items were included to provide a brief self‐assessment and, although not psychometrically validated, were reviewed during pilot testing for clarity and relevance to occupational therapy practice.

Perceptions of AI were measured with the Shinners Artificial Intelligence Perception (SHAIP) tool (Shinners et al., 2023), a validated 10‐item instrument with two subscales: Professional Impact of AI and Preparedness for AI. General AI literacy was assessed using the AI Literacy Scale (Pinski & Benlian, 2023), a 28‐item tool covering five dimensions of socio‐technical competencies. The survey concluded with four open‐ended questions on AI platforms used, perceived barriers and facilitators, and training needs.

### 2.4 Data collection and analysis

Surveys were completed anonymously online using the REDCap platform (Harris et al., 2009), with paper copies available on request. Paper surveys were distributed in staff meetings and returned anonymously before being entered into REDCap. All responses were subsequently exported to Stata for analysis (StataCorp, 2025).

Quantitative data were analysed using descriptive and exploratory inferential statistics for group comparisons with frequencies, percentages, means, and standard deviations. Group comparisons were completed to explore whether perceptions varied by clinical context, professional experience, or prior exposure to AI. Occupational therapy and AI experience were reported in years, and service settings were grouped into acute, sub‐acute/community, and aged care to support robust analysis.

Chi‐square tests were used to examine associations between categorical variables, and ANOVA was used to compare mean scale scores across groups. Although individual Likert items are ordinal, SHAIP and AI Literacy Scale scores represent summed multi‐item measures and were treated as continuous variables, consistent with prior validation studies. Assumptions for ANOVA were assessed by the inspection of residual distributions and homogeneity of variance using Levene's test. Expected cell counts were examined for chi‐square analyses; where assumptions were not met, Fisher's exact tests were considered. Spearman's correlation coefficients were calculated to examine associations between age and self‐rated AI knowledge, skills, confidence, and comfort.

Descriptive content analysis of open‐ended responses (Vaismoradi et al., 2013) generated categories relating to current AI use, perceived barriers, facilitators, and training needs. Two researchers independently coded responses, compared coding structures, and resolved discrepancies related to the categories by consensus. Frequencies were reported to indicate the relative prominence of categories.

## 3 FINDINGS

Fifty‐three therapists completed the survey (response rate 44%), the majority of whom were female (n = 51, 96%). The mean age was 29.4 years (SD = 10.3, range 23–59, median 28, IQR 25–34), and they averaged 7.3 years of practice (SD = 6.3, range <1–23, median 5, IQR 3–12). AI experience was minimal (M = 0.47 years, SD = 0.75, range 0–3, median 0, IQR 0–1). Respondents worked across sub‐acute/community (n = 25, 47%), acute (n = 21, 40%), and aged care (n = 10, 19%) settings, with smaller numbers in rapid response, paediatrics, and hand therapy services.

### 3.1 AI knowledge, skills, confidence, and comfort

**FIGURE 1:**
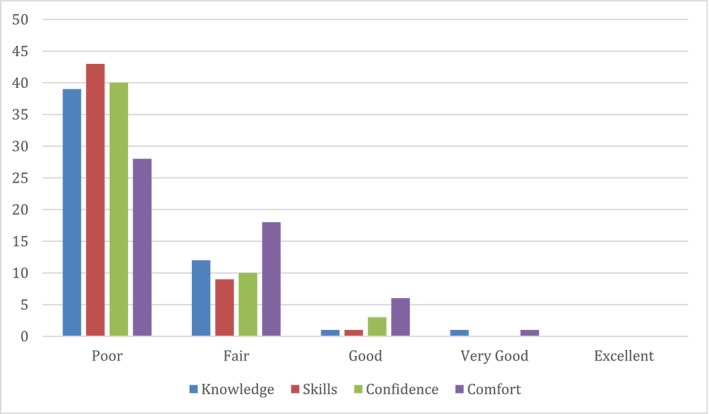
Number of participants rating AI knowledge, skills, confidence, and comfort.

Participants rated AI knowledge (M = 1.32), skills (M = 1.21), confidence (M = 1.30), and comfort (M = 1.62) as poor to fair (Figure 1).

### 3.2 Perceptions of AI (SHAIP)

On the SHAIP, participants perceived AI as having the potential to improve patient care and decision making but reported a lack of preparedness for its implementation. The lowest scoring item was related to training in AI use specific to occupational therapy. Awareness of workplace ethical frameworks was also limited (Table 1).

**TABLE 1:** Shinners Artificial Intelligence Perception (SHAIP) tool scores.

I believe that …	M (SD)
AI in OT could improve the delivery of patient care	3.51 (0.67)
AI in OT could improve clinical decision making	3.08 (0.83)
AI can improve population health outcomes	3.45 (0.72)
AI will change my role as a health‐care professional in the future	3.58 (0.89)
AI will reduce financial cost associated with my role	3.13 (0.76)
Health‐care professionals are prepared for the introduction of AI	2.96 (1.07)
AI may take over part of my role as an OT	2.25 (0.92)
I am adequately trained to use AI specific to OT	1.81 (0.92)
My workplace has ethical frameworks in place for AI	2.64 (0.88)
Full responsibility for AI errors lies with the OT	3.11 (0.97)

Abbreviation: OT, occupational therapy.

### 3.3 AI literacy

AI Literacy Scale scores were generally low, particularly for technical knowledge like AI processing methods and information representation. However, the understanding of human roles within AI collaboration was higher. Experience items indicated generally minimal engagement with AI technologies, including limited interaction with different AI platforms and almost no experience designing or developing AI systems (Table 2).

**TABLE 2:** AI Literacy Scale scores.

I have knowledge of …	M (SD)
The technology AI is built on	1.72 (0.79)
How AI and non‐AI technology differ	1.79 (0.88)
OT cases of AI implementation	1.57 (0.69)
Roles of AI in H/AI interaction	1.66 (0.76)
Who (excluding programmers) enables H/AI collaboration	1.60 (0.69)
What AI does better than humans	2.00 (0.81)
What humans do better than AI	2.40 (0.93)
Who sets up and manages H/AI collaborations	1.58 (0.63)
Human tasks within H/AI collaborations	1.72 (0.77)
AI input data requirements	1.58 (0.77)
How AI perceives input data	1.55 (0.72)
Potential impacts of input data has on AI	1.66 (0.73)
Input data types for AI	1.49 (0.70)
AI processing methods and models	1.38 (0.60)
Information representation for AI processing	1.40 (0.60)
AI processing risks	1.91 (0.81)
AI processing as a learning process	1.75 (0.81)
Using and interpreting AI output	1.68 (0.83)
Limitations of AI output	1.81 (0.88)
Handling AI output	1.60 (0.74)
AI outputs currently obtainable	1.51 (0.70)

Rate current experience	M (SD)
Interacting with different types of AI	2.06 (1.03)
Frequent AI use in everyday life	1.81 (0.90)
Designing AI models	1.08 (0.27)
Developing AI products	1.06 (0.23)

Overall AI literacy	M (SD)
I know the unique roles of AI and humans in H/AI collaboration	1.89 (0.99)
I know the steps involved in AI decision making	1.62 (0.79)
I am relatively proficient in the field of AI	1.58 (0.82)

Abbreviations: H/AI, human/artificial intelligence; OT, occupational therapy.

### 3.4 Perceptions of the professional impact of AI

Over half reported current use of AI platforms, mainly ChatGPT (n = 15) and Siri/Alexa (n = 5). Participants generally agreed that AI could improve the delivery of patient care (M = 3.51), improve population health outcomes (M = 3.45), and improve clinical decision making (M = 3.08). They anticipated that AI would change their professional role in the future (M = 3.58) but expressed moderate agreement that AI may take over part of their role (M = 2.25). Perceived financial cost reduction associated with AI was moderate (M = 3.13).

Qualitative findings supported these perceptions. Participants identified potential benefits, including improved efficiency, support for documentation, enhanced evidence use, and reduced paperwork. However, barriers included limited knowledge (n = 21), privacy and ethical concerns (n = 10), uncertainty about alignment with person‐centred practice (n = 6), and concerns about reliability. Respondents questioned whether AI could adequately address holistic patient needs.

### 3.5 Influence of service setting, years of experience, or prior AI exposure

Group comparisons were conducted using service setting (acute, sub‐acute/community, and aged care), years of occupational therapy experience, and prior AI exposure as independent variables. No statistically significant differences were identified across service setting, years of experience, or prior AI exposure for SHAIP total or subscale scores. Similarly, no significant group differences were found for AI Literacy Scale scores. These findings indicate that levels of AI knowledge, literacy, confidence, comfort, and preparedness were consistently low across therapist groups within this service.

### 3.6 Relationship with AI knowledge, skills, confidence, and comfort

Strong positive correlations were observed between knowledge and skills (r = 0.72, P < 0.001) and between skills and confidence (r = 0.71, P < 0.001). Moderate correlations were identified between comfort and knowledge (r = 0.43, P < 0.01), skills (r = 0.40, P < 0.01), and confidence (r = 0.53, P < 0.001). These findings indicate that therapists who reported greater knowledge also reported greater skills, confidence, and comfort with AI.

## 4 DISCUSSION

This study explored occupational therapists' perceptions of AI in an Australian tertiary health service, focussing on its potential, workforce preparedness, and training needs. Overall, occupational therapists reported low AI literacy and preparedness but moderate optimism regarding AI's potential professional impact. Perceptions did not differ significantly across service setting, years of experience, or prior AI exposure. Knowledge, skills, and confidence were strongly associated, suggesting interconnected dimensions of AI literacy within this workforce.

Low AI literacy aligns with broader health‐care findings. Kokkotis et al. (2025) noted limited evidence for discipline‐specific applications, which may explain occupational therapists' low engagement. Occupational therapists acknowledged AI's potential but reported little direct experience, reflecting the gap between technological advances and clinical uptake. Other studies (Catalina et al., 2023; Matharu et al., 2025; Tezpal et al., 2024; Vanamali et al., 2025) similarly show health‐care professionals eager for tailored training to close this gap.

Positive attitudes towards AI match those in the general population, which tends to support health‐care AI while remaining cautious (Horst et al., 2025; Yakar et al., 2022; Young et al., 2021). Derakhshanian et al. (2024) also found health science students supportive yet concerned about job security. Our finding that knowledge, skills, and confidence were strongly correlated mirrors their observation of links between exposure, attitudes, and utilisation intent. Years of occupational therapy experience did not influence AI knowledge or attitudes, challenging assumptions that early career clinicians are automatically more comfortable with technology (Cinalioglu et al., 2022).

Occupational therapists in this study held moderate concerns about AI replacing occupational therapists' jobs. Canadian occupational therapists also reported fewer fears about job loss than other professions (Matharu et al., 2025). Although occupational therapy is considered low risk for automation (Liu, 2018), occupational therapists expressed greater concern about privacy and client‐centred care issues, suggesting that the occupational therapy context differs from other health professions and requires nuanced training approaches.

These findings are very relevant to the professional challenges mentioned in Section 1 regarding critical engagement with AI, understanding its ethical and professional limits, and effective implementation in the real world. Low confidence and AI literacy limit the ability of occupational therapists to critically appraise AI outputs and increase the risk of over‐reliance on AI systems (Akhtar et al., 2025; Hoffman et al., 2024; Vanamali et al., 2025). The findings also indicate that there are gaps in understanding the purpose, use, and limitations of AI systems and their ethical implications for client‐centred practice (Kaelin et al., 2024; Shinners et al., 2023). However, desire from occupational therapists for more examples of AI applications relevant to occupational therapy indicates that they want to upskill around the use of AI for their practice (Kokkotis et al., 2025; Stover & Jacobs, 2025). This indicates workforce development needs to combine both technical capacity building (i.e., prompt design) with content addressing critical thinking and applied ethics grounded in examples from everyday practice.

### 4.1 Strengths and limitations

The use of validated tools (SHAIP and AI Literacy Scale) ensured robust measurement of perceptions, whereas the mixed‐methods design supported comprehensive insights. Limitations include the small, single‐service sample, reliance on self‐report, and cross‐sectional design, which restrict generalisability and causal inference. The lack of significant group differences may reflect limited statistical power or indicate that meaningful differences do not exist, suggesting that future studies with larger, more diverse cohorts are warranted.

### 4.2 Implications for practice, policy, research, and education

Within this service, the findings suggest a need for targeted AI training programmes addressing basic technical concepts, such as data processing and limitations, as these scored lowest on the AI Literacy Scale. Given occupational therapists' uncertainty about workplace frameworks, services may benefit from establishing and communicating clear policies defining clinician responsibility for AI use. These findings also suggest that integrating AI literacy into occupational therapy education curricula may help prepare students and early career occupational therapists for evolving workplaces (Stover & Jacobs, 2025). Case‐based workshops demonstrating occupational therapy‐specific applications may be one approach to addressing the current lack of hands‐on experience.

Future research should adopt longitudinal approaches to track AI adoption and test interventions, generating evidence to guide broader uptake. Collaborative work with consumers and interdisciplinary teams will be critical for ensuring AI tools reflect real‐world needs and enhance occupational engagement.

## 5 CONCLUSIONS

This study shows that occupational therapists in an Australian tertiary health service hold a positive yet cautious view of AI. Despite their optimism, they reported low literacy and preparedness, indicating a need for targeted education and clear policies. Addressing these needs will help ensure that AI strengthens, rather than diminishes, the unique contribution of occupational therapy to human health and supports the continuing evolution of the profession.

## AUTHOR CONTRIBUTIONS

Claire del Rio, Helen Nelson, and Danielle Hitch collaboratively contributed to the conception and design of the study; acquisition, analysis, and interpretation of data; drafting and revising the manuscript critically for intellectual content; and final approval of the version to be published. Each author agrees to be accountable for all aspects of the work, ensuring accuracy and integrity.

## CONFLICT OF INTEREST STATEMENT

The authors declare that there are no conflicts of interest in relation to this work.

## ETHICS STATEMENT

Ethics approval for the study was obtained from the Western Health Human Ethics Committee (QI663). Participation was voluntary, and all participants provided assumed informed consent when submitting the anonymous survey. No identifying information was collected, and data were stored securely.

## CONSUMER AND COMMUNITY INVOLVEMENT

There was no consumer and community involvement in this study.

## USE OF ARTIFICIAL INTELLIGENCE (AI)‐ASSISTED TECHNOLOGY

Generative artificial intelligence (AI) tools were used only to support writing clarity and language editing during manuscript preparation. No AI technology was used to generate intellectual content, data, or analysis, and all authors take full responsibility for the integrity and originality of the work.

## Data Availability

Data are available from the authors upon reasonable request.
